# Is psychologically vulnerable rural-to-urban migrants' mental health further at stake under China's tightened COVID-19 measures: how should the government respond?

**DOI:** 10.3389/fsoc.2023.1095810

**Published:** 2023-05-05

**Authors:** Jason Hung

**Affiliations:** Department of Sociology, The University of Cambridge, Cambridge, United Kingdom

**Keywords:** mental health, internal migrants, COVID-19, pandemic, health policies

## Abstract

There is a well-documented scholarly discourse on how the COVID-19 outbreak adversely affects the mental health of Chinese nationals. However, there is little evidence revealing how the public health crisis negatively influenced the mental health of rural-to-urban migrants in China. The relevant literature argues that rural-to-urban migrant workers and their dependents have been experiencing discrimination, exclusion, and stigmatization in the urban labor market and social space, thereby jeopardizing their mental health to a large extent. However, whether the COVID-19 outbreak and its associated consequences further compounded rural-to-urban migrant workers' mental health has rarely been discussed. Since 2010, the Chinese government has emphasized the importance of promoting positive mental health. Without identifying how COVID-19 specifically imposed mental health challenges on rural-to-urban migrant cohorts, Chinese policymakers cannot effectively and efficiently address the dilemmas faced by such vulnerable groups. The significant rural-to-urban migrant population (i.e. 291 million), alongside their disposition to encounter social and psychological challenges, prompts the urgency to develop this narrative essay to examine whether their mental health burdens worsened during the pandemic. The author also discusses remedies for the worsened mental issues faced by migrant cohorts, and recommends policies that local Chinese governments should adopt to mitigate the mental health burdens encountered by rural-to-urban migrants.

## 1. Introduction

In December 2019, the coronavirus (COVID-19) outbreak occurred in Wuhan, China. The crisis escalated to a public health emergency on 21st January, 2020 (Song et al., 2020). Since then, the Chinese government had employed strict public health measures, including locking down the epicenter of the outbreak, applying nationwide travel restrictions, and exercising home quarantine (Liu et al., 2021). There is a well-documented scholarly discourse on how the COVID-19 outbreak adversely affects the mental health of Chinese nationals (e.g., Li et al., 2020; Ren et al., 2020; Zhou et al., 2020). However, there is little evidence revealing how the public health crisis negatively influenced the mental health of rural-to-urban migrants in China.

The relevant literature argues that rural-to-urban migrant workers and their dependents have been experiencing discrimination, exclusion, and stigmatization in the urban labor market and social space, thereby jeopardizing their mental health to a large extent (Hung, 2019; Yang et al., 2021). Supporting literature shows that rural-to-urban migrants suffered from more mental health issues (such as depression) than their native urban or rural counterparts, possibly because their disproportional encounters with deprivation and socioeconomic discrimination and disadvantages (Li and Rose, 2017). However, whether the COVID-19 outbreak and its associated consequences further compounded rural-to-urban migrant workers' mental health has rarely been discussed. Since 2010, the Chinese government has emphasized the importance of promoting positive mental health. A pre-COVID-19 estimate suggests that 16.6 percent of Chinese nationals suffered from some form of mental health challenges (Fang et al., 2020). To maintain a sustainable healthcare system and human resources supply, the Chinese Government has, for example, prioritized the development of mental health facilities, such as spending a total of 184.28 million USD on building psychiatric hospitals in 2018, compared to 44.58 million USD in 2009 (Fang et al., 2020; Jia et al., 2021). These figures demonstrate how heavily the Chinese government has valued the promotion of mental health and delivery of psychological and psychiatric support in recent years.

Without identifying how COVID-19 specifically imposed mental health challenges on rural-to-urban migrant cohorts, Chinese policymakers cannot effectively and efficiently address the dilemmas faced by such vulnerable groups. In 2019, there were 291 million rural-to-urban migrant laborers in China, comprising 36 percent of the entire working population nationwide (China Labour Bulletin, 2021). The significant rural-to-urban migrant population, alongside their disposition to encounter social and psychological challenges, prompts the urgency to develop this narrative essay to examine whether their mental health burdens worsened during the pandemic. The author also discusses remedies for the worsened mental issues faced by migrant cohorts, and therefore, recommends policies that local Chinese governments should adopt to mitigate the mental health burdens encountered by rural-to-urban migrants.

## 2. Mental health challenges during the pandemic

Within China, the rapid dissemination of COVID-19 was due to the substantial number of Chinese rural-to-urban migrant workers returning to villages or towns during the Spring Festival. In 2020, for example, from 10th January to 24th January, over 1.14 billion trips by train were recorded. Those returning to rural areas underwent 2 weeks of quarantine. Compulsory and lengthy quarantine measures, along with the economic pressure imposed by the waves of COVID-19-related unemployment, had resulted in notable mental health issues among Chinese migrant laborers (Liu et al., 2020). Quarantine and lockdown often exposed individuals to a wide range of stressors, such as loneliness, depression, fatigue, insomnia, suicidal attempts, and a fall in subjective well-being (Jia et al., 2021). Worrying about COVID-19 infection or actual infection compounded the mental health burden among such cohorts.

Liem (2021) suggests that rural-to-urban migrants suffer from a higher risk of developing psychological challenges, such as anxiety and depression during the pandemic, relative to their urban native counterparts. This is because rural-to-urban migrants are more socially isolated and marginalized in cities. In addition, they were entitled to fewer social protection, welfare, and insurance packages when residing in cities, given that many urban social benefits have been exclusively designated for local urban cohorts (Liem, 2021). Since the 1980s, major rural-to-urban migration destinations such as Shanghai and Guangzhou have been delivering a growth of urban healthcare welfare and benefits. Under such policies, Shanghai and Guangzhou have been experiencing a surge in outpatient psychiatry visits, a growth in popularity of mental health hotlines and radio call-in programmes, and a rising utilization of available pharmaceutical agents and psychological counseling services that have disproportionately been accessible to better-educated and wealthier urban elites. Rural-to-urban migrant laborers, however, have been discriminated against and deprived from accessing the majority of these urban healthcare benefits (Chang and and Khleinman, 2002). Therefore, the lack of social connections was more likely to bar rural-to-urban migrants from accessing social or community support in cities, whether they or their dependents contracted COVID-19 or experienced financial hardships. However, there is an absence of literature unveiling whether rural-to-urban migrants are financially capable of providing unsubsidized social and health insurance. Future research should focus on assessing whether better-skilled, better-educated, and better-paid rural-to-urban migrants were more socially protected relative to their less-skilled, educated counterparts, as either their work units would subsidize COVID-19 related social benefits or they could afford unsubsidized social support. Future research should also examine whether rural-to-urban migrants living with their dependents are inclined to develop more mental health burdens relative to their migrant counterparts who live alone. This is because those living with their dependents might worry about contracting COVID-19 and passing the disease to their family members. Rural-to-urban migrant workers who occupied less-skilled, more physically demanding jobs often performed hard labor and could not work remotely to avoid COVID-19 infection. These hard laborers might be at a further risk of encountering mental health burdens.

According to a report named *the China Enterprise Reform and Development Society*, the pandemic imposed more adverse impacts on employees of private enterprises than on those of state-owned enterprises. Employees working in private enterprises are less satisfied with their job conditions and have higher turnover intentions (Song et al., 2020). Employees who feel less secure occupationally experience more mental health burdens owing to the concern that they might be unemployed and lose their source of income (Song et al., 2020). In China, the labor market comprises state-owned enterprises, private enterprises, and the agricultural sector. State-owned enterprises offer the highest wage levels and socio-occupational security benefits for employees, followed by private enterprises and agricultural businesses (Fields and Song, 2013). Lower-skilled rural-to-urban migrant laborers are least likely to secure jobs in state-owned enterprises. However, better-educated and skilled migrants and local urban laborers have access to most state-owned enterprises' job opportunities (Akgüç et al., 2014). These arguments suggest that less-educated and less-skilled rural-to-urban migrant workers were exposed to a higher risk of facing socio-occupational insecurity and mental health challenges directly or indirectly caused by the pandemic. Here, an additional year of schooling raises a migrant's opportunity for employment by 2.5–3.5 percent (Yueping et al., 2021). These statistics support the understanding that better-educated rural-to-urban migrants were less affected by the COVID-19-related economic predicament. Their possession of educational credentials served, to some degree, as a safety net to ensure their socio-occupational security, and thus mitigate, their encounters with mental issues.

Aside from social exclusion, cultural and linguistic isolation could also drive rural-to-urban migrant workers to develop mental health problems. Liem (2021) noted that migrants who lack Mandarin fluency suffered from a higher risk of anxiety and depression in the early stages of the pandemic. Perhaps, such a finding shows that encounters with language barriers discouraged migrant cohorts from seeking necessary social, health, or community support during the COVID-19 epoch, compounding the vulnerability and sense of helplessness among Chinese migrant workers. It is noteworthy that, in the pre-pandemic epoch, 20.1 percent of Chinese migrant workers experienced depression, which was nearly four times (5.9 percent) higher than their local rural residents (Liu et al., 2020). The figures suggest that internal migration *per se* is associated with confrontation with significant psychological stressors. Prior to the outbreak of the pandemic, a portion of psychologically vulnerable rural-to-urban migrants might have already been on the verge of a mental breakdown. The occurrence of the pandemic, along with the emergence of its associated economic consequences, might have significantly traumatized the well-being of these psychologically fragile cohorts. With unsatisfactory Mandarin proficiency, they experienced difficulties in communication and information access, exacerbating their predicament of being socially and informationally isolated and excluded.

Public health and economic uncertainties, alongside the emergence of misinformation about COVID-19 on social media, deepened the experiences of anxiety, depression, and sleep disturbance among rural-to-urban migrants and Chinese nationals at large (Liu et al., 2020). However, Liu et al. (2020) failed to indicate whether rural-to-urban migrants developed more adverse psychological symptoms than Chinese nationals. Future research should comparatively analyze the mental health challenges faced by rural-to-urban migrants, urban natives, and rural natives amid the pandemic, for the purpose of indicating if rural-to-urban migrants were relatively particularly vulnerable. If so, local governments should form more mental health interventions designated for rural-to-urban migrant cohorts to ensure that such vulnerable cohorts could weather storms in the post-pandemic epoch.

Rural-to-urban migrants have often been criticized as suspected carriers of diseases and criminals in the pre-pandemic epoch (Chakraborty, 2020). In particular, when misinformation on social media is rampant, migrant cohorts may be further targeted as COVID-19 carriers. Future research should examine how such xenophobia adversely affects the mental health of rural-to-urban migrants under a public health emergency. COVID-19-related xenophobia might heighten the social isolation and exclusion faced by rural-to-urban migrants, compounding their inferior status in cities and harming their subjective sense of security while residing in urban areas.

In this section, the author summarizes how social, occupational, and linguistic isolation or insecurity faced by rural-to-urban migrant cohorts might heighten their encounters with mental health challenges during the pandemic. As there are only a handful of assessments of rural-to-urban migrant cohorts' mental health during the public health crisis, the author suggests some research directions that relevant scholars could consider shall they aim to discover whether and how rural-to-urban migrants' mental health worsened during the pandemic. In the following section, the author briefly discusses some remedies to improve internal migrants' mental health.

## 3. Remedies for better mental health

Fortunately, most rural-to-urban migrants own smartphones, an instrument that can be used to seek informational and social support in the post-pandemic epoch (Liem et al., 2020). The Chinese government regularly disseminates public health updates and information on WeChat, a popular Chinese social networking platform. Rural-to-urban migrants can use WeChat or other social media to receive health messages and obtain emotional support from village-based family members via texting or video calling. Rural-to-urban migrants who experience social isolation in cities can capitalize on their telecommunication devices to seek connections with their families and friends in order to mitigate their lack of subjective well-being and sense of loneliness.

Since the pandemic, the Chinese government has set up a freely accessible “tele-mental health services” system, allowing any Chinese nationals to receive psychological support, such as digital counseling (Chakraborty, 2020). Such a system minimizes the risk of COVID-19 infection and enables the nationwide mass delivery of mental health support, including for those living in remote, less-facilitated neighborhoods. However, those who are digitally (semi-)illiterate may not be able to enjoy these services.

As mentioned, rural-to-urban migrants are excluded from the entitlement to a raft of social welfare designated for urban natives. These migrant cohorts, especially those who are less educated and skilled, are unlikely to be able to afford private, unsubsidized social and health insurance (Liu et al., 2020). Therefore, the delivery of “tele-mental health services” can serve as an alternative means for rural-to-urban migrants to receive basic healthcare support on a non-discriminatory basis. The provision of these freely accessible services is particularly beneficial to socially and financially vulnerable cohorts, including migrants.

## 4. Policy recommendations

The author briefly addresses existing remedies for promoting the mental health of rural-to-urban migrants. Despite COVID-19 no longer being deemed a pandemic, China remains one of the countries that continues to apply strict public health measures to combat local outbreaks. Given China's prudent stance, in this section, the author will recommend policies that China may consider undertaking in order to ensure that rural-to-urban migrants' mental health is not overlooked.

Under a public health emergency, local governments in China should rearrange their social and healthcare services. To date, many services provided in cities have been designated for urban natives. The exclusion and discrimination faced by rural-to-urban migrants placed them in a notably vulnerable position in the post-pandemic epoch. Until a public health emergency is lifted, local governments should allow rural-to-urban migrants to claim a fairer share of social and health insurance packages in cities (Liem et al., 2020; Qiu et al., 2020). If they or their dependents are infected with COVID-19, they should be permitted to enjoy subsidized welfare packages for healthcare services and social protection that are currently designated for urban natives. Here more state-subsidized social and healthcare services should be distributed on a non-discriminatory basis. Not only should rural-to-urban migrant laborers in the formal sector be entitled to the access to the welfare, but those working in the informal sector—who are not protected by labor contracts—should also be given the opportunities to benefit the use of state-subsidized social and healthcare benefits. Such a suggested interim policy will be conducive to the promotion of inclusivity in today's public health system, thereby ensuring that no vulnerable cohorts will be left behind in anti-COVID-19 protection.

To cope with the public health crisis, China-leading mental health institutions and academic communities have developed guidelines to promote individuals' mental health, including “the Manual of Mental Health Services during the COVID-19 Outbreak (Liu et al., 2020)”. Chinese officials should translate these guidelines into reader-friendly materials for laypersons, particularly those who are (semi-)illiterate. They should translate these textual guidelines into audio and visual forms, further enabling individuals who lack literacy to understand. Based on these guidelines, a range of psychological services, including online mental health education and 24-h hotlines, has been established in many Chinese regions (Liu et al., 2020). The provision of these services should be extended to remote, least-developed Chinese areas. As Chinese nationals in different regions, especially villages, use a wide variety of Chinese dialects or ethnic minority languages, the hotliners on duty should be able to communicate in the dialects or languages that the callers primarily speak. Such a policy helps break the linguistic barriers that discourage rural-to-urban migrant cohorts from accessing psychological support services.

Moreover, in China, ample booths have been built, and the health specialists on duty are responsible for performing COVID-19 tests on local residents on a regular basis. Additional specialists should be assigned to booths to deliver mental health-screening services to local residents. Here, specialists should provide a written or oral form of a mental health screening survey to local residents of interest. For survey respondents who are deemed at a high risk of mental health challenges, specialists should refer the cases to one of the nearby psychological or psychiatric professionals for follow-up. Alternatively, for survey respondents who are viewed as being at moderate risk of having mental health issues, specialists should pass the cases to one of the nearby social workers for follow-up. Mass mental health screening enables the identification of individuals, including rural-to-urban migrants, in need of psychological support, and therefore, ensures access to prompt healthcare services.

## 5. Conclusion

As mentioned, since 2010, the Chinese government has prioritized the promotion of better mental health to build more sustainable futures. Despite such a focus on Chinese national policy, in the pre-pandemic epoch, the mental health of rural-to-urban migrants was overlooked to some degree owing to their inferior status. However, the COVID-19 outbreak and China's decision to continually tighten its public health measures have harmed the well-being of rural-to-urban migrants. If rural-to-urban migrants' mental health challenges are not addressed appropriately and in a timely manner, the presence of mentally disordered rural-to-urban migrants will become a significant liability to any host city. This is because the productivity of mentally ill rural-to-urban migrants is plausibly limited, and unproductive, or even unemployed, rural-to-urban migrants rely on local governments' basic unemployment support to make ends meet. To ensure that rural-to-urban migrants can continually be seen as valuable labor and assets rather than liabilities, under the public health emergency, the Chinese government should focus more on the provision of psychological and psychiatric support to migrant cohorts in need.

## Author contributions

JH was solely responsible for designing, writing, editing and submitting this mini review for publication consideration.
